# A New Sign of Pregnancy

**Published:** 1846-06

**Authors:** 


*A new sign of Pregnancy.*—By Dr. Pollenden.—The author states, that during a practice of eighteen years he had observed a peculiar smell of the vaginal mucus to be a constant and unerring sign of pregnancy. The smell is musty, like that of spermatic fluid or liquor amnii; and, after a vaginal examination, it cannot be mistaken for any other odour. In a great many cases of pregnancy, during the first, second, and third months, when the condition of the patient was doubtful, owing to the earliness of the period, the author never in a single instance failed to discover the true state of the party by means of this sign. According to his latest observations the odour is perceptible as early as the eighth day of gestation.—*Northern Journal of Medicine*, from *Med. Correspondenzblatt Rhein.*

